# Health Disparities in Hospitalized Pediatric Patients with Autism Spectrum Disorder and COVID-19

**DOI:** 10.3390/children11111363

**Published:** 2024-11-09

**Authors:** Janet Lee, Lisa Ferretti, Camden Nelson, Priya Nigam, Jessica Zawacki, Philip McCallion

**Affiliations:** 1 Lewis Katz School of Medicine, Temple University, Philadelphia, PA 19122, USA; 2 School of Social Work, College of Public Health, Temple University, Philadelphia, PA 19122, USA; 3 ABA Centers of America, Nashua, NH 03063, USA

**Keywords:** autism spectrum disorder, COVID-19, health disparities

## Abstract

Background/Objectives: Pediatric patients with autism spectrum disorder (ASD) face unique challenges, especially amongst individuals from historically minoritized racial groups. ASD has also been associated with an increased mortality from COVID-19. This study aims to explore the differences in sociodemographic factors and health outcomes (as measured by length of stay) amongst hospitalized pediatric patients with COVID-19 infections and a diagnosis of ASD compared to individuals with a COVID-19 infection alone; Methods: We performed a retrospective cohort study examining pediatric patients (ages birth to 21) who were hospitalized with a diagnosis of ASD and COVID-19 compared to patients with a diagnosis of COVID-19 alone between January 2019 and June 2023 using Epic Systems Corporation’s Cosmos, a de-identified dataset aggregated from electronic health record data. We examined differences in demographic factors and length of stay (LOS) between groups by utilizing chi-square and Wilcoxon rank sum tests. Multiple logistic regression models were utilized to assess the association between length of stay and diagnosis; Results: A total of 21,708 distinct pediatric patients with a diagnosis of ASD and COVID-19 or COVID-19 alone were included in the analytical dataset. Patients with ASD and COVID-19, compared to patients with COVID-19 alone, had a higher proportion of individuals identifying as male and White. Patients with COVID-19 alone, compared to individuals with ASD and COVID-19, had higher proportions of individuals identifying as Black or African American. Higher proportions of individuals with ASD and COVID-19 had public insurance, compared to individuals with COVID-19 alone. Having a diagnosis of ASD and COVID, after controlling for covariates, was associated with higher odds of having a length of stay greater than the three days (cutoff value determined by the median LOS of three days) compared to having a diagnosis of COVID alone (aOR 1.19, 95% CI 1.04–1.35); Conclusions: Our study highlights the health disparities experienced during hospitalizations by pediatric patients with ASD and COVID-19. Further studies should address barriers and support health outcomes for pediatric patients with ASD.

## 1. Introduction

Autism spectrum disorder (ASD) is neurodevelopmental disorder. To meet the diagnostic criteria for ASD according to the DSM-5, a child must have persistent deficits in each of the three areas of social communication and interaction plus at least two of the four types of restricted, repetitive behaviors [1]. ASD is diagnosed across all racial, ethnic, and socioeconomic groups, affecting an estimated 1 in 36 children; however, it is almost four times more likely in males [2]. Prior to the COVID-19 pandemic, studies have demonstrated population level differences in the prevalence of ASD amongst children. There was a higher prevalence of ASD in children with higher socioeconomic status compared to those with lower socioeconomic status (SES). Additionally, there was a higher prevalence of ASD in children who identified as non-Hispanic White, compared to children who identified as Hispanic or non-Hispanic Black. These findings were not fully explained by racial disparities in SES but could be indicative of the underascertainment of ASD in these populations [3]. While historically more commonly identified in Caucasian children, there has recently been a shift in epidemiology, with 2020 data indicating for the first time that African American and Hispanic children are more likely to be diagnosed [4,5]. This could be attributed to increased efforts to universally screen children in the United States for ASD [6].

The COVID-19 pandemic had far-reaching impacts on numerous communities. Having comorbidities (i.e., hypertension, diabetes, cardiovascular disease, etc.) increased the likelihood of having severe COVID-19 and mortality [7]. Communities that were comprised of less than 25% of individuals who identified as Caucasian had the highest odds of hospitalization associated with COVID-19 in 2020 [8]. Neighborhoods in the state of New York with higher proportions of African American and Hispanic residents had higher COVID-19 infection rates per capita [9].

Individuals with ASD are more likely to have comorbid conditions, particularly with gastrointestinal, metabolic, and immunological disorders and higher health costs [10]. Additional health disadvantages experienced by patients with ASD include limitations on insurance coverage, high deductibles, decreased understanding of public health guidance, and lack of healthcare provider competence in neurodiversity [6]. These unique circumstances may have contributed to individuals with ASD having higher odds of hospitalizations, mortality, and longer hospital stays related to COVID-19 than those without ASD [8,10]. Hospitalizations and increased length of stay (LOS) for patients with ASD were further increased with co-occurring intellectual and developmental delays [8,10]. Children and adults with ASD are particularly vulnerable to infections, including COVID-19, given contacts due to their higher dependence on services from outside caregivers, higher odds of living in congregate settings, increased co-morbidities at baseline, difficulties with preventative guidelines, and challenges in recognizing and communicating health issues [8,11].

Though the relationship between ASD and hospitalizations for COVID-19 has been demonstrated in the literature, our understanding of the disparities in hospitalized children with ASD and COVID-19 is not fully understood. We would expect that children with ASD and COVID-19 would have worse health outcomes, given their reliance on caregivers, communication challenges, and comorbid conditions, including immunological disorders [12]. This study aims to explore the differences in sociodemographic factors and health outcomes amongst hospitalized pediatric patients with COVID-19 infections and a diagnosis of ASD compared to individuals with a COVID-19 infection alone. We hypothesized that children with ASD and COVID-19 would have an increased likelihood of longer hospital stay compared to children hospitalized with COVID-19 alone after adjusting for sociodemographic variables and comorbidities.

## 2. Materials and Methods

### 2.1. Study Sample and Setting

We performed a retrospective cohort study examining pediatric patients (ages birth to 21) who were hospitalized with a diagnosis of autism spectrum disorder (ASD) and COVID-19 compared to patients with a diagnosis of COVID-19 alone between January 2019 and June 2023 using Epic Systems Corporation’s Cosmos [12]. Cosmos is a HIPAA-defined limited dataset created in collaboration with a community of Epic health systems representing more than 257 million patient records from 1548 hospitals and more than 34,600 clinics from all 50 states and Lebanon [12]. Notably, different health systems participating in the Cosmos network attribute data at different times to the Cosmos dataset. Data within Cosmos was originally collected within contributing health systems’ electronic health records (EHR). EHR data may have several limitations, including inaccurate or incomplete data, and lack of availability of measures related to behavioral health [13]. Given the limitations of the dataset, patients with missing data were not included in the analytical dataset.

Inclusion criteria included any pediatric patient (ages 0–21) who had a hospitalization for COVID-19 with and without a diagnosis of ASD between January 2019 and June 2023. Diagnoses for ASD were identified by aggregating the following ICD-10-CM codes: F84.0 (autistic disorder); F84.3 (other childhood disintegrative disorder); F84.5 (Asperger’s syndrome); F84.8 (other pervasive developmental disorders); and F84.9 (pervasive developmental disorder, unspecified). Hospitalizations were defined as patients who were seen in the emergency department and/or admitted to the inpatient unit. Exclusion criteria included any patients who were missing study measure data.

This study was deemed to not constitute human subjects research and did not require IRB review or approval.

### 2.2. Study Measures

Study variables included BMI, length of stay (LOS) for the hospitalization, and sociodemographic variables. Notably, BMI percentiles were not available for analysis in this dataset. Length of stay was the outcome variable chosen to measure severity of illness associated with COVID-19 infection. Sociodemographic variables were selected a priori based on availability within Cosmos and social determinants of health associated with health outcomes. Sociodemographic variables included race, ethnicity, social vulnerability index (SVI), and financial class.

Social vulnerability index (SVI), a variable used to describe the demographic and socioeconomic factors that contribute to communities being more adversely affected by public health stressors that cause disease and injury [14], and financial class (i.e., insurance type) were utilized as proxies for social context. The SVI measure within the Cosmos dataset is as a continuous variable with values between 0 and 1, with higher values indicating a higher degree of social vulnerability [14]. SVI was transformed from a into a categorical variable, grouped by SVI quartile to improve interpretability of values. In this study, we defined individuals with SVI quartiles 3 and 4 as more socially vulnerable, and SVI quartiles 1 and 2 as less or socially vulnerable.

The financial class variable is a measure indicating patients’ insurance type. Financial class had multiple subcategories with low counts (i.e., unspecified, self-pay, Medicare), which were aggregated into one group entitled, “Miscellaneous/Other”. This aggregation was done in accordance with Epic’s Cosmos privacy and security policies [12]. The “Medicaid” insurance subcategory represented individuals who were on government sponsored or public insurance. The “Miscellaneous/Other” group represented individuals who had private insurance.

In accordance with Epic’s Cosmos privacy and security policies [12], Asian, Native Hawaiian, and Pacific Islander groups racial groups was aggregated into one group entitled, “AAPINH”. Policy has recently supported the disaggregation of race data for individuals from the Asian American and Native Hawaiian/Pacific Islander communities [15], but because of the low counts of individuals who identified as either Native Hawaiian or Pacific Islander, these groups were aggregated for this study.

### 2.3. Statistical Analyses

Descriptive statistics were used to determine median and interquartile range for continuous variables between pediatric patients hospitalized with ASD and COVID-19 compared to the COVID-19 only group, while frequency and percentages were utilized for categorical variables. Bivariate analyses were performed using chi-square tests to evaluate differences between categorical variables, inclusive of demographic and socioeconomic factors (i.e., sex, SVI, race, ethnicity, and financial class). Wilcoxon rank sum tests were used to compare differences in our non-normally distributed dataset between groups for continuous variables (i.e., age, BMI, length of stay).

A multivariable logistic regression model was fitted to assess the association between length of stay and diagnosis controlling for sociodemographic covariates). Variables included in the multivariable model were determined in univariate logistic regression models (i.e., race, SVI, age, financial class). We reported odds ratios (ORs) for the unadjusted logistic regression model and adjusted odds ratios (aORs) according to 95% confidence intervals (CIs). *p*-values of 0.05 were considered statistically significant. Data were analyzed in the R environment (R version 4.2.3, http://www.r-project.org, accessed on 28 September 2024) [16].

## 3. Results

### 3.1. Descriptive Statistics

A total of 25,250 distinct pediatric patients with a diagnosis of autism spectrum disorder (ASD) and COVID-19 or COVID-19 alone were identified within Cosmos. Cases that were missing BMI, discharge date, SVI, financial class, and race data were excluded from the dataset, meaning 21,708 cases were included in the analytical dataset. Table 1 presents the descriptive statistics of the total sample. A total of 4.7% (*n* = 1028) of patients had a diagnosis of ASD and COVID-19, compared to 95% (*n* = 20,680) having a diagnosis of COVID-19 alone. The median age of the patients was 11 years old (IQR 5–16). 49% (*n* = 10,667) of the population identified as female, while 51% (*n* = 11,041) identified as male. 58% (*n* = 12,483) of the total population identified as White, 28% (*n*= 6059) of the total population identified as African American or Black, and 23% (*n* = 4905) of the population identified as Hispanic. Of the total population, 62% (*n* = 13,478) had a financial class or insurance type of miscellaneous/other, while 38% (*n* = 8230) of patients had a financial class or insurance type of Medicaid (government sponsored insurance). The median length of stay of hospitalization was three days (IQR 2–6). Of the total hospitalizations, 70% (*n* = 15,213) started in the emergency department.

### 3.2. Sociodemographic Differences Between Patients with ASD and COVID-19 Compared to COVID-19 Alone

Table 2 highlights sociodemographic differences between individuals in the ASD and COVID group and the COVID only group. Chi-square tests of independence were performed to examine the relation between diagnosis (i.e., ASD + COVID vs. COVID alone) and categorical sociodemographic factors. In the ASD + COVID group, 69% (*n* = 710) of patients identified as male, compared to the COVID only group in which 50% (*n* = 10,331) of patients identified as male. The relationship between diagnosis and sex was significant, *X*^2^ (1, *n* = 21,708) = 142.33, *p* < 0.0001, effect size 0.08. Adjusted standard residuals demonstrated that the difference between groups was likely related to a higher proportion of patients in the ASD + COVID group identifying as male.

**Figure 1:**
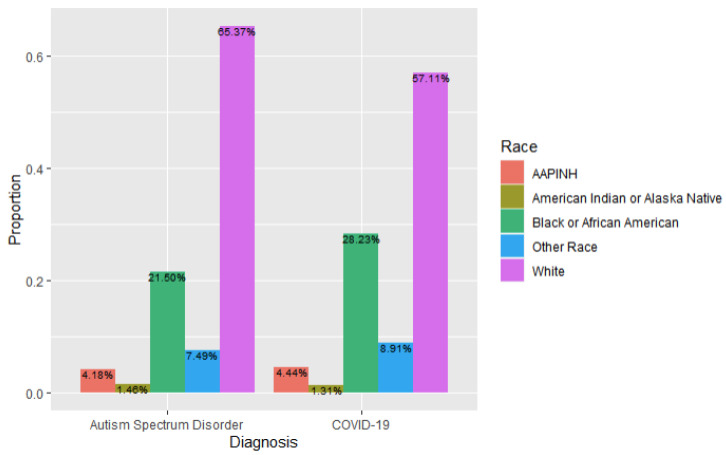
Race Breakdown by Diagnosis Type (ASD and COVID-19 versus COVID-19 Alone).

In the ASD + COVID group, 65% (*n* = 672) of individuals identified as White, while 57% (*n* = 11,811) of individuals in the COVID only group identified as White. A total of 21% (*n* = 221) of individuals in the ASD + COVID group identified as Black or African American, compared to 28% (*n* = 5838) in the COVID only group. The relation between diagnosis and race was significant, *X*^2^ (4, *n* = 21,708) = 30.07, *p* < 0.001, effect size = 0.019, likely related to a higher proportion of subjects in the ASD and COVID group identifying as White, and a higher proportion of patients in the COVID only group identifying as African American/Black (Figure 1).

In the ASD + COVID group, 18% (*n* = 181) of individuals identified as Hispanic or Latino, compared to 23% (*n* = 4724) of individuals in the COVID only group. The relation between diagnosis and ethnicity was significant, *X*^2^ (2, *n* = 21,708) = 20.18, *p* < 0.001, effect size = 0.022, likely related to a higher proportion of individuals in the COVID group identifying as Hispanic.

**Figure 2:**
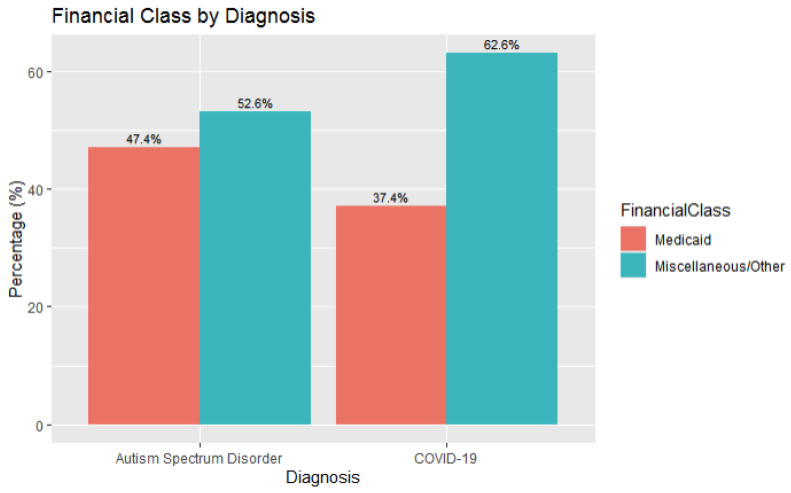
Financial Class Breakdown by Diagnosis Type (ASD and COVID-19 versus COVID-19 Alone).

In the ASD + COVID group, 47% (*n* = 487) of patients had Medicaid (government sponsored) insurance, compared to 37% (*n* = 7743) of patients in the COVID only group having Medicaid insurance. The relation between diagnosis and financial class, as defined by insurance type, was significant, *X*^2^ (1, *n* = 21,708) = 40.616, *p* < 0.001, effect size 0.04, and is likely related to a higher proportion of patients in the ASD and COVID group having Medicaid insurance (Figure 2).

Higher SVI quartiles (i.e., 3 and 4) are associated with higher levels of social vulnerability. In the ASD + COVID group, 53% (*n* = 545) patients had SVI quartiles in groups 3 and 4, compared to the COVID only group which had 57% (*n* = 11,940) of patients with SVI quartiles in groups 3 and 4. In the ASD + COVID group, 47% (*n* = 483) had SVI quartiles in groups 1 and 2, compared to the COVID only group which 42% (*n* = 8740) had SVI quartiles in groups 1 and 2. The relationship between diagnosis and social vulnerability index (categorized into quartiles) was significant, *X*^2^ (3, *n* = 21,708) = 23.184, *p* < 0.001, effect size = 0.02, likely related to higher proportions of subjects in the COVID group having higher SVI quartiles. The relation between diagnosis and whether the hospitalization started in the emergency department was not significant, *X*^2^ (1, *n* = 21,708) = 0.0461, *p* < 0.83.

Wilcoxon rank sum tests were performed to examine the relationship between diagnosis (i.e., ASD and COVID vs. COVID alone) and continuous variables (i.e., age, body mass index). The median age in the ASD and COVID group was 12 years (IQR 7–16), whereas the median age in the COVID only group was 11 years (IQR 4–16). The Wilcoxon test showed that the difference was significant (*p* < 0.001, effect size r = 0.056). There were no significant differences in BMI between the ASD and COVID and COVID only groups.

### 3.3. Relationship Between Diagnosis and Length of Stay

Multiple logistic regression models were used to examine the relationship between length of stay (LOS) and diagnosis (please refer to Table 3). Length of stay served as our outcome variable to measure the severity of illness associated with COVID-19 infection. A cut-off value of LOS of greater or less than three days was chosen based on the median LOS for the total population. In the univariable model, having a diagnosis of ASD and COVID was associated with 1.28 times higher odds of having a LOS greater than three days, compared to the COVID only group (OR 1.28, 95% CI 1.12–1.45). After controlling for covariates (age, sex, race, ethnicity, financial class, SVI quartile), having a diagnosis of ASD and COVID was associated with 1.19 higher odds of having a length of stay greater than three days, compared to those who had a diagnosis of COVID alone (aOR 1.19, 95% CI 1.04–1.35). Interaction terms between age and diagnosis, sex and diagnosis, race and diagnosis, ethnicity and diagnosis, and insurance and diagnosis did not significantly moderate the effect.

### 3.4. Relationship Between Covariates and Length of Stay

In addition to diagnosis, five covariates in the multivariable logistic regression model were associated with a LOS greater than three days. Older age groups (14–18 years old) were associated with 2.57 times higher odds of having a LOS greater than three days while controlling for covariates (aOR 2.57, 95% CI 2.39–2.78) compared to the reference group of children aged zero to four years old. Identifying as female, compared to male, was associated with 1.06 times higher odds of having a LOS greater than three days while controlling for covariates (aOR 1.12, 95% CI 1.01–1.12).

Identifying as AAPINH, Black, or African American was associated with an increased odds of having a LOS greater than three days, compared to those who identified as White. Identifying as AAPINH was associated with 1.23 times higher odds of having a LOS greater than three days compared to those who identified as White (aOR 1.23, 95% CI 1.07–1.41). Identifying as Black or African American was associated with having 1.14 times higher odds of having a LOS greater than three days, compared to those who identified as White (aOR 1.14, 95% CI 1.06–1.22). Identifying as Other Race was associated a lower likelihood of having a LOS greater than three days, compared to who identified as White (aOR 0.87, 95% CI 0.79–0.97).

Identifying as Hispanic or Latino, while controlling for covariates, was associated with 1.13 times higher odds of having a LOS greater than three days, compared to those who did not identify as Hispanic or Latino (aOR 1.13, 95% CI 1.04–1.21). Having Medicaid insurance, while controlling for covariates, was associated with 1.17 times higher odds of having a LOS greater than three days, compared to those who had Miscellaneous or Other insurance (aOR 1.17, 95% CI 1.11–1.24).

## 4. Discussion

This study described differences in sociodemographic factors and health outcomes, as characterized by longer hospital stays, amongst hospitalized pediatric patients with COVID-19 infections and a diagnosis of autism spectrum disorder (ASD), compared to individuals in the same age group with a COVID-19 infection alone. This study considered the role of sociodemographic factors and supported the hypothesis that pediatric patients with ASD and COVID-19 had higher odds of a longer hospitalization compared to patients with COVID-19 alone. Longer hospitalizations are typically associated with poorer health outcomes. Thus, the results of this study maybe suggest that pediatric patients with ASD may experience greater risk for worse health outcomes associated with COVID-19. Prior studies looking at health outcomes associated with ASD and COVID-19 infection demonstrated similar results with an increased odds of longer hospitalization in patients with ASD and COVID-19 compared to COVID-19 alone but did not focus explicitly on the pediatric population [10,17].

Worse health outcomes in patients with ASD and COVID-19 may be explained by differing immunological phenotypes, reliance on caregivers, communication challenges, and comorbid conditions that may play a role in increased morbidity [10,17]. Psychosocial factors, including societal exclusion, could potentially exacerbate existing disparities [10,17].

Higher proportions of pediatric patients with a diagnosis of ASD and COVID-19 were likely to identify as male and White, and less likely to identify as Hispanic compared to patients with COVID-19 alone. Our findings differed from prior literature that demonstrated that ASD prevalence was decreasing among non-Hispanic White children [18], pointing to possible effective policy measures to increase universal screening for ASD in children [5,18,19]. These differences could be related to data collection differences across studies [5,18], including self-report, surveillance data, and provider entered electronic health record ICD-10 codes for ASD diagnosis [5,18]. It is also possible that the Cosmos dataset did not completely capture ASD diagnosis data. Our findings were consistent with prior studies that demonstrated higher rates of COVID-19 associated hospitalizations amongst individuals who identified as non-Hispanic Black or African American [20].

Compared to prior studies, our population had higher proportions of patients with diagnoses of ASD and COVID-19 having Medicaid insurance compared to patients with COVID-19 alone (47% vs. 37%). Our findings were consistent with prior studies which demonstrated that children with ASD had higher proportions of public insurance (1 in 4) compared to those with private insurance (1 in 9) [21]. Our study looked specifically at hospitalizations, while prior studies examined population-level data unrelated to COVID-19 infection [21]. This difference could have contributed to higher levels of patients in our cohort who had public insurance. Medicaid, or public insurance, could be interpreted as a proxy for socioeconomic status. Interestingly, the prior literature demonstrated that children with ASD were more likely to have a higher socioeconomic status [3]. It is possible that the complex interplay of ASD, poverty, sociodemographic health factors, and COVID-19 could have widened the already existing health disparities for pediatric patients with ASD and contributed to a higher likelihood of longer hospitalization.

Patients in our study with a diagnosis of ASD and COVID-19 had lower social vulnerability (SVI) indices compared to individuals with COVID-19 alone. Though SVI quartiles were lower for pediatric patients with ASD, overall, a higher proportion of individuals with ASD and COVID had higher SVI quartiles (53%, *n* = 545) compared to lower SVI quartiles (47%, *n* = 483). This highlights that individuals who were hospitalized for COVID-19 who also had a diagnosis of ASD were more likely to be socially vulnerable. The prior literature has shown higher socioeconomic status to be associated with an increase in ASD prevalence [3]. Similar to our findings around to public insurance, it is possible that the dynamic factors of ASD, poverty, social vulnerability, and COVID-19 could have amplified existing health disparities, leading to an increase in hospitalizations for COVID-19 in individuals with ASD.

BMI has been an important factor in health outcomes associated with COVID-19), where higher BMI levels and obesity were associated with worse health outcomes [17,22,23]. Additionally, ASD in pediatric patients has been associated with higher BMI measurements and diagnoses for obesity [24]. Interestingly, there were no significant differences in BMI between pediatric patients with ASD and COVID compared to COVID alone. Notably, BMI percentile data was not available for analysis in the Cosmos dataset.

### Limitations

Our study was innovative in that it utilized a large, de-identified data set generated from electronic health data to better understand differences between our study groups. Our study population was diverse across race, ethnicity, and socioeconomic status and is largely similar to the population described by United States Census data [12]. Though health disparities have been noted in pediatric patients with ASD, our study specifically demonstrated that patients with diagnoses of ASD and COVID-19, after controlling for various factors, had a higher likelihood of longer hospitalizations, compared to pediatric patients with COVID-19 infections alone. Non-pediatric specific studies have demonstrated similar results with longer hospitalizations and a higher likelihood of hospitalization in patients with ASD and COVID-19 [10,17].

Though the Cosmos dataset is large and representative of the United States population, there were many cases that had to be removed secondary to missing data [11]. Removing missing data could have introduced omitting missing variable bias into our analytical dataset, which could impact the interpretability and generalizability of our findings. Data aggregated in Cosmos is generated from EHR data. There could be many limitations to the dataset, including factors contributing to how data is collected and how clinical decisions are made [13]. The authors of this study worked to utilize clinical knowledge to inform selection of data elements to combat these challenges. Despite thoughtful aggregation of ICD-10 codes was employed to query the Cosmos dataset, sample selection bias could have affected our results [13].

Further limitations of EHR data include possible inconsistencies in documentation of certain data elements. Our dataset was also limited by which factors were available from the EHR. Thus, we were not able to collect additional information around factors such as behavioral health support, family structure, psychosocial support, and urban versus rural settings. Because Cosmos is a de-identified dataset, zip code or geographical data was not available for analysis. We were also not able to collect data around BMI percentiles, which is the preferred measure in pediatric populations [24]. Given the limitations of the dataset and Cosmos privacy and security policies [12], data for individuals from the Asian and Native Hawaiian/Pacific Islander communities were aggregated for this study. The aggregation of racial data limits the ability to draw specific conclusion for individuals in these racial groups. Future efforts should be made to disaggregate racial groups to better understand the complex needs of diverse communities. Finally, because of de-identification requirements for low count data, financial class/insurance type categories had to be aggregated for analysis. This limits our ability to draw specific conclusions around how insurance status might have impacted our results.

## 5. Conclusions

Our study demonstrates the health disparities experienced during hospitalizations by pediatric patients with ASD and COVID-19. When caring for patients with ASD, providers and organizations should consider the many complex factors that may affect health outcomes. Further studies around potential drivers of these disparities should be conducted.

Targeted interventions should be geared towards supporting the complex psychosocial and medical needs of pediatric patients and families with ASD. These interventions could include improved multidisciplinary mental health and behavioral services and collaboration for individuals with ASD. Given the growing number of patients with ASD and public insurance, policies should support increasing access to multidisciplinary services and medical care for patients with Medicaid insurance. Policymakers should allocate additional resources to support clinical care and research around pediatric patients with ASD. Furthermore, further studies on the long-term impacts of the COVID-19 pandemic on the health and well-being of pediatric patients with ASD should be conducted.

## Figures and Tables

**Table 1:** Total Population Characteristics (*n* = 21,708).

Characteristics	*n* = 21,708 ^1^
Age in Years	11 (5.0, 16.0)
Sex	
Female	10,667 (49%)
Male	11,041 (51%)
Race	
AAPINH	961 (4.4%)
American Indian or Alaska Native	285 (1.3%)
Black or African American	6059 (28%)
Other Race	1920 (8.8%)
White	12,483 (58%)
BMI	17 (14, 21)
Ethnicity	
Hispanic or Latino	4905 (23%)
Not Hispanic or Latino	16,803 (77%)
Financial Class	
Medicaid	8230 (38%)
Miscellaneous/Other	13,478 (62%)
Length of Stay	3.0 (2.0, 6.0)
Started in ED	15,213 (70%)
Diagnosis	
Autism Spectrum Disorder	1028 (4.7%)
COVID-19	20,680 (95%)
SVI Quartile	
1	4697 (22%)
2	4526 (21%)
3	5294 (24%)
4	7191 (33%)

^1^ Median (IQR); n (%).

**Table 2:** Population Characteristics by Diagnosis Type (ASD and COVID-19 versus COVID-19 Alone).

Characteristics	Autism Spectrum Disorder, *n* = 1028 ^1^	COVID-19, *n* = 20,680 ^1^	*p*-Value ^2^
Age in Years	12 (7.0, 16.0)	11 (4.0, 16.0)	<0.001
Sex			<0.001
Female	318 (31%)	10,349 (50%)	
Male	710 (69%)	10,331 (50%)	
Race			<0.001
AAPINH	43 (4.2%)	918 (4.4%)	
American Indian or Alaska Native	15 (1.5%)	270 (1.3%)	
Black or African American	221 (21%)	5838 (28%)	
Other Race	77 (7.5%)	1843 (8.9%)	
White	672 (65%)	11,811 (57%)	
BMI	17 (14, 22)	17 (14, 21)	0.10
Ethnicity			<0.001
Hispanic or Latino	181 (18%)	4724 (23%)	
Not Hispanic or Latino	847 (82%)	15,956 (77%)	
Financial Class			<0.001
Medicaid	487 (47%)	7743 (37%)	
Miscellaneous/Other	541 (53%)	12,937 (63%)	
Length of Stay	3.5 (2.0, 7.0)	3.0 (2.0, 5.0)	<0.001
Started in ED	724 (70%)	14,489 (70%)	0.8
SVI Quartile			<0.001
1	284 (28%)	4413 (21%)	
2	199 (19%)	4327 (21%)	
3	238 (23%)	5056 (24%)	
4	307 (30%)	6884 (33%)	

^1^ Median (IQR); n (%), ^2^ Wilcoxon rank sum test; Pearson’s Chi-squared test.

**Table 3:** Univariable and Multivariable Logistic Regression Models for Diagnosis Type on Length of Stay.

			Univariable			Multivariable	
Characteristics	N	OR ^1^	95% CI ^1^	*p*-Value	OR ^1^	95% CI ^1^	*p*-Value
ASD	21,485						
False		__	__		__	__	
True		1.28	1.12, 1.45	<0.001	1.17	1.03, 1.33	0.017
Age	21,485						
0–4		__	__		__	__	
5–8		1.50	1.37, 1.64	<0.001	1.48	1.35, 1.62	<0.001
9–13		2.18	2.00, 2.38	<0.001	2.16	1.98, 2.35	<0.001
14–18		2.60	2.42, 2.81	<0.001	2.57	2.39, 2.78	<0.001
19–22		2.16	1.90, 2.46	<0.001	2.15	1.89, 2.45	<0.001
Sex	21,485						
Male		__	__		__	__	
Female		1.12	1.07, 1.19	<0.001	1.06	1.01, 1.12	0.032
Race	21,485						
White		__	__		__	__	
AAPINH		1.07	0.93, 1.22	0.3	1.23	1.07, 1.41	0.003
American Indian or Alaska Native		1.12	0.88, 1.42	0.3	1.03	0.81, 1.31	0.8
Black or African American		1.15	1.08, 1.22	<0.001	1.14	1.06, 1.22	<0.001
Other Race		0.91	0.82, 1.00	0.055	0.87	0.79, 0.97	0.015
Ethnicity	21,485						
Not Hispanic or Latino		__	__		__	__	
Hispanic or Latino		1.04	0.97, 1.11	0.2	1.13	1.04, 1.21	0.002
SVI Quartile	21,485						
SVI ≤ 2		__	__		__	__	
SVI ≥ 2		1.03	0.98, 1.09	0.2	0.97	0.92, 1.03	0.3
Financial Class	21,485						
Miscellaneous/Other		__	__		__	__	
Medicaid		1.18	1.12, 1.25	<0.001	1.17	1.11, 1.24	<0.001

^1^ OR = Odds Ratio, CI = Confidence Interval.

## Data Availability

The original contributions presented in this study are included in the article. The datasets presented in this article are not readily available because the datasets are maintained by the Epic Systems Corporation. Requests to access the datasets should be directed to the Epic Systems Corporation.
